# ‘Therapy or no therapy, I’m still going to have unusual experiences [ … ] but it has [helped] the way that I feel and cope.’ A qualitative exploration of service user experiences of EMDRp and TF-CBTp in the ‘REducing psychosiS risk by TARgeting Trauma‘ study

**DOI:** 10.3389/fpsyt.2026.1812693

**Published:** 2026-09-01

**Authors:** Aidan Flinn, Filippo Varese, Marina Sandys, Amanda Larkin, Kim Cartwright, Clare Holden, Samantha Bowe, David Keane, Debbie Malkin, Diane Griffiths, Christopher D. J. Taylor, Sophie Parker, Kate Allsopp

**Affiliations:** 1 Division of Psychology and Mental Health, School of Health Sciences, Manchester Academic Health Science Centre, University of Manchester, Manchester, United Kingdom; 2 Complex Trauma and Resilience Research Unit, Greater Manchester Mental Health NHS Foundation Trust, Manchester Academic Health Science Centre, Manchester, United Kingdom; 3 Independent Researcher, Manchester, United Kingdom; 4 Lancashire and South Cumbria Traumatic Stress Service, Lancashire and South Cumbria NHS Foundation Trust, Chorley, United Kingdom; 5 Mersey Care NHS Foundation Trust, Prescot, United Kingdom; 6 Pennine Care NHS Foundation Trust, Ashton-under-Lyne, United Kingdom; 7 School of Psychology, University of Sheffield, Sheffield, United Kingdom; 8 Youth Mental Health Research Unit, Greater Manchester Mental Health NHS Foundation Trust, Manchester Academic Health Science Centre, Manchester, United Kingdom

**Keywords:** at-risk mental state for psychosis, clinical high risk, qualitative, trauma, ultra-high risk

## Abstract

**Introduction:**

Traumatic experiences are common and exert substantial influence on clinical outcomes for individuals with an at-risk mental state for psychosis (ARMS). Despite a growing quantitative literature, no study has systematically examined ARMS service users’ qualitative perspectives on acceptability and feasibility of trauma-focussed interventions. The present study is the first to address this gap by exploring the views of ARMS participants who took part in the RESTART Trial, a feasibility trial comparing up to 24 sessions of EMDRp or TF-CBTp with Treatment as Usual.

**Methods:**

A total of 22 ARMS participants completed semi-structured interviews. Data were analysed using the National Centre for Social Research Framework approach, guided by the Theoretical Framework of Acceptability. Purposeful sampling captured a wide range of demographic backgrounds and treatment experiences.

**Results:**

Participants noted that both EMDRp and TF-CBTp were experienced as safe, effective, and acceptable. High perceived effectiveness, particularly for symptoms associated with post-traumatic stress, were reported. While reductions in ARMS experiences were noted, participants more often described a shift in their relationship to symptoms, gaining a greater sense of power and control. Participants demonstrated a clear understanding of the interventions and felt they aligned with their personal values. Although emotional burden and treatment demands were acknowledged, participants recommended some individually tailored adaptations to improve their impact.

**Discussion:**

Our findings highlight the potential value of trauma-focussed interventions for ARMS, demonstrating that such therapies are viewed as acceptable, meaningful, and impactful. Larger-scale trials are needed to establish definitive clinical effectiveness, clarify mechanisms of change, and inform clinical guidelines.

## Introduction

The link between traumatic life events and the risk of developing severe mental health difficulties is well established. Meta-analytic evidence suggests that individual and cumulative exposure to traumatic events in childhood significantly increases the odds of experiencing adult psychosis (1, 2). Traumatic events are similarly pervasive in young people and young adults experiencing an ‘At-Risk Mental State’ (ARMS), with prevalence rates as high as 86.8% (3). Traumatic events are an independent predictor of ARMS status (4), and specific exposure of emotional and physical abuse has been significantly linked to an increased odds of experiencing an ARMS (5). Individual studies have highlighted the link between any exposure (6) and specific events (e.g., sexual and physical abuse (7); and ARMS symptomology. Exposure has also been linked to significant co-morbidities, such as cognitive impairments, affective symptoms, and suicidality (8–11). As such, recent reviews have highlighted the critical need to target and treat the impact of traumatic events in ARMS populations to improve clinical outcomes (12).

To address trauma-based needs in these populations, attention has turned to establishing an evidence base for targeted interventions such as Eye Movement Desensitization and Reprocessing (EMDR) and Trauma Focused-Cognitive Behavioural Therapy (TF-CBT). Both therapies have a significant evidence base for targeting traumatic symptoms, recommended by the National Institute of Health and Care Excellence (NICE), with research indicating reductions in psychotic symptoms in first episode populations (13, 14). However, further work is needed, and is currently ongoing, to adapt these interventions to the needs of people with psychosis. Emphasis on collaborative formulation, relational safety, and careful preparation (such as an extended stabilisation phase) has led to the development of EMDR and TF-CBT for psychosis (EMDRp, TF-CBTp (15, 16)). A recently completed feasibility randomised control trial (RCT) has indicated efficacy and acceptability of up to 16 sessions of EMDRp in participants with psychosis (17), and other trauma-focussed approaches have been effective in reducing psychotic sequalae in a recent review (18). However, these positive developments have not led to the same level of innovation for those experiencing an at-risk mental state for psychosis. Early promising findings have been provided by a feasibility RCT comparing eight sessions of EMDR, to a waitlist condition, finding significant reductions in both traumatic and ARMS symptomology (19). However, further research needs to be completed on larger samples and to consider other established trauma focussed interventions to ensure that the trauma-related needs of ARMS individuals are addressed and supported by a robust evidence base.

Qualitative explorations of the views of psychosis and ARMS individuals on trauma and trauma-focussed interventions are limited. For those diagnosed with psychosis, individuals noted that disclosure, while difficult, was a net positive and outlining a strong link between their current difficulties and previous adversities (20). A strong desire for trauma-focussed treatment has also been noted, but significant barriers existed, leading to exclusion from services (21). A single mixed-methods study has explored the viewpoints of ARMS individuals who had received trauma-focussed Therapy (EMDRp) as part of a smaller trial (22). Participants who completed EMDRp highlighted the effectiveness of reducing symptoms, whereas a significant number of non-completers noted no effect and feeling overwhelmed by the emotions associated with therapy. However, this study had a small sample size, a significant number of non-completers, and did not focus on specific acceptability outcomes or potential adaptions needed for this population group. This highlights the need for further studies to explore the views of these populations.

The present article was a qualitative study nested within a feasibility RCT exploring the acceptability of EMDRp and TF-CBTp in the ARMS population (23). The aim of the current sub-study was to gather a qualitative understanding on factors related to the acceptability and feasibility of trauma-focussed interventions for ARMS individuals who took part in the RESTART trial. The study also gathered information related to participants’ understanding of their own experiences, and guide adaptations to the therapies and trial procedures to inform further definitive trials.

## Methods

### Design and theoretical framework

The study involved qualitative semi-structured interviews informed by a modified version of the Sekhon’s Theoretical Framework of Acceptability (24), comprising seven key constructs illustrated in **Table 1**.

**Table 1:** Sekhon’s acceptability framework and definitions operationalised for the present study.

Construct	Definitions
Affective attitude	What were participants’ attitudes towards their trauma history, therapeutic support, and the links between adversities and their current experiences.
Intervention coherence	To what extent did participants understand how trauma-focussed interventions work (e.g., what are stages of the intervention, how does the intervention help them meet their goals)?
Perceived effectiveness	To what extent did participants feel trauma-focussed interventions were effective for them?
Burden	To what extent did participants think trauma-focussed interventions would cause some form of burden (emotional, practical)? What factors did participants feel interfered with their ability to participate in these interventions?
Opportunity costs	To what extent did participants feel they needed to make compromises within their lives to effectively engage with trauma-focussed interventions?
Self-efficacy	How confident were participants that they could engage with trauma-focussed interventions?
Ethicality	To what extent did participants see trauma-focussed interventions as fitting within their values? Would any adaptations need to be made for these interventions to better fit participants’ values?

### Participants

Participants were individuals who took part in the REducing psychosiS risk by TARgeting Trauma’ (RESTART) trial. RESTART was a feasibility randomised controlled trial, with three arms (EMDRp + Treatment As Usual (TAU), TF-CBTp + TAU, or TAU alone). The study aimed to assess the feasibility and acceptability of up to 24 sessions of trauma-focussed interventions for those assessed to be experiencing an at-risk mental state for psychosis. RESTART, as the parent study, recruited 76 participants (ISRCTN76699092). Further details on the participant journey can be seen in the published protocol paper (23).

A recruitment target of 18 to 24 was deemed to be sufficient for the qualitative study, given the use of established theoretical frameworks and sample specificity (25). Recruitment ended after 22 participants after it was deemed that data saturation was achieved. This decision was made following reports from the first author that no new themes were being identified in the interviews and then discussed and agreed within the senior research team (AF, KA, and FV).

All trial participants who expressed interest in the qualitative interview were approached to take part after their final follow-up, which commenced immediately after the end of their allocated treatment window. Participants were eligible if they (i) took part in the feasibility RCT and (ii) had capacity and willingness to provide informed consent. Eligibility for the feasibility RCT required individuals to be (i) over the age of 16, (ii) meeting ARMS criteria as defined by the Comprehensive Assessment of At-Risk Mental States (CAARMS-23), and (iii) reported at least one exposure to a potentially traumatic life event as defined by the Trauma and Life Event Checklist (TALE). Participants were excluded from RESTART, and thus the present study, if they were (i) requiring an interpreter to take part in the intervention, (ii) either assessed by the research team or previously as experiencing a first episode of psychosis and/or previous or current treatment with antipsychotics at dose of over 5 mg of haloperidol or equivalent for over 3 weeks, or (iii) judged by the assigned care coordinator/responsible clinician and the research team as not sufficiently clinically stable to engage safely in a clinical trial of trauma-focussed therapy.

Participants were purposively sampled for maximum variation to ensure a diverse sample in terms of key demographic characteristics (age, gender, ethnicity, and neurodiversity), trial arm, completeness of the intervention, variable trauma histories, and ARMS symptoms. Researchers actively sought out participants with differing experiences to ensure their views were represented. This included a participant who withdrew from the intervention and one who transitioned to a first episode of psychosis (FEP).

### Data collection

Interviews were conducted from September 2024 to September 2025 and were completed by first author and lived experience researcher AF (n=20) alongside research worker MS (n=2) who are both trained and experienced in qualitative research data collection methods. Interviews primarily took place online or via the phone (n=19), as per participant preference, with all participants providing either written or recorded informed consent, alongside completing a demographic questionnaire. Semi-structured interviews were audio-recorded using an encrypted dictaphone and transcribed verbatim by either an external transcription service or a member of the research team. Interviews were pseudo-anonymised at point of transcription.

Interviews were completed utilising purposefully designed topic guides, depending on allocation, based on the acceptability concepts defined above. The full topic guides, with question labels, can be seen in **Supplementary Materials 1, 2**. In brief, the topic guides consisted of five sections seeking information related to (i) participants’ feelings before being referred to the trial, (ii) experiences of support and/or intervention while under the trial, (iii) specific therapy feedback (if applicable), (iv) trial participation, and (v) future trial feedback. Participants allocated to TAU were interviewed using a shortened guide, removing section iii, due to the lack of intervention provided by the trial. Interviews were designed to last between 45 and 90 min. Changes to the topic guides according to participant and research input were discussed within the senior research team (KA and AF) and adjusted accordingly.

As the interview was completed in a semi-structured fashion, consistency in topics was achieved, while also allowing for the interviewer to be guided by participants’ responses. The flexibility of this approach was explained to participants, and they were given freedom to discuss any relevant topic that they felt appropriate. It was made clear in the approach and throughout the interview that the team was keen to hear feedback about aspects of the therapy or trial procedures that they felt could be improved or were not so helpful. Participants were explicitly encouraged to share these experiences and reassured that their feedback would not be identifiable to their therapists or research workers; they were advised that feedback could be used to inform procedures of a future trial. Participants were also asked if they would like to receive a copy of their interview transcripts and a summary of emerging findings for the study, to allow for ‘member checking’. There were 12 participants (54.55% of the sample) who requested a copy of their interview transcripts and emerging findings. All participants confirmed receipt, and no changes were suggested as a result.

### Data analysis

Data were coded in NVivo 14 by the first author (AF) with the support of senior researcher KA. AF and KA met regularly throughout the analysis process to discuss and resolve any discrepancies.

The National Centre for Social Research ‘Framework’ analysis approach (26) was used to analyse the transcripts, deductively coded according TFA constructs. Framework analysis was chosen due to the systematic approach to analysing qualitative data. Additional themes of importance were derived inductively. In line with framework analysis steps, indexing was completed according to the responses to the questions designed to address the construct (**Supplementary Materials 1, 2**). Charting was completed in NVivo 14 to ensure that the summaries were traceable to the raw data. Cross-arm comparisons were conducted by the researchers by reviewing individual answers to intervention specific question to identify any differences in experiences of therapies. To ensure accuracy, participants who requested copies of their transcripts were invited to comment on how the findings resonated with their experiences and to ensure that they feel they were reflected in the collected data.

### Lived experience input

Experts by experience, from the Lived Experience Advisory Panel at the Complex Trauma and Resilience Research Unit (C-TRU), were involved throughout the study. Group members provided feedback on the overall study design and topic guides for the project alongside the emerging and finalised themes following completion of the interviews. The interviews were also primarily conducted by lived experience researcher AF, who utilised their own experience to inform the topic guide and interviews when appropriate.

### Ethical approval

Ethical approval for the study was granted by West Midlands – South Birmingham Research Ethics Committee (REC), REC reference 23/WM/0113 on 22nd May 2023; Health Research Authority, IRAS ID 323676.

## Results

A total of 22 participants were included in the final analysis. Seven trial participants declined to take part, primarily due to participant perceived ongoing social stressors unrelated to the study. Following discussion with the wider RESTART team, and based on the information available, there was no indication that these individuals had significantly different experiences than those interviewed. Nine of the interview participants received Trauma-Focused CBTp, eight received EMDRp, and five were allocated Treatment As Usual. A single participant withdrew from the trial assessments (TF-CBTp arm) but agreed to complete the interview. Participants who were allocated to a therapy arm completed a mean of 20.76 (SD = 4.48) sessions and had a wide range of traumatic experiences and ARMS symptoms that were focussed on in therapy. Participants had an average age of 26.5, and 86.36% were white. There were 20 participants who reported the same gender identity assigned at birth, whereas 16 self-reported either a diagnosis provided by a clinical team external to the trial or self-identified as neurodiverse (including but not limited to autism, ADHD, and dyslexia). Average demographics of the trial were similar to those of the main trial. Information about trial allocation and pre-trial therapy experiences can be seen in **Table 2**. The final themes and findings were reviewed by the Patient and Public Involvement and Engagement (PPIE) group within the research unit, and authors AF, KA, and FV. Final codes included all acceptability constructs alongside the inductive codes of ‘Therapeutic Relationship’, ‘Participant Buy-In’, ‘Therapeutic Adaptions’, and ‘Limitations of TF Therapy’. For the purposes of the current study, key findings from these themes are encompassed in the Perceived Effectiveness, Affective Attitude, and Ethicality sections, respectively. Further quotes can be found in **Table 3**, and any comment in the text in italics is taken as a direct quote from a participant.

**Table 2:** Information about trial and pre-trial therapy experiences.

Pseudonym	Allocated group	Number of sessions (if allocated to treatment)	Previous experiences of therapy
SU_01	TAU	–	CBT with EDIT^a^
SU_02	TF-CBTp	14	CBT with EDIT
SU_03	EMDRp	21	CBT with EDIT
SU_04	TF-CBTp	16	Counselling under CAMHS
SU_05	TF-CBTp	22	CBT with another clinical trial and EDIT
SU_06	EMDRp	24	CBT through EDIT
SU_07	TAU	–	CBT and general counselling
SU_08	TF-CBTp	8	General counselling
SU_09	TF-CBTp	24	Counselling under CAMHS
SU_10	EMDRp	22	General counselling
SU_11	TAU	–	Private counselling
SU_12	TAU	–	CBT from IAPT
SU_13	TF-CBTp	20	General counselling
SU_14	TF-CBTp	24	None
SU_15	TF-CBTp	24	None
SU_16	EMDRp	24	General counselling
SU_17	EMDRp	24	CBT and general counselling
SU_18	TAU	–	EMDRp and general counselling
SU_19	TF-CBTp	23	CBT with EDIT
SU_20	EMDRp	24	CBT with EDIT
SU_21	EMDRp	16	CBT with EDIT
SU_22	EMDRp	23	CBT with EDIT

^a^EDIT, Early Detection and Intervention Team.

**Table 3:** Additional quotes.

Concept	Quote
Affective Attitude	*I had a safe space to express my emotions and someone who could understand them better than I ever could- SU_04* *No, I didn’t feel rushed, everything was quite – I could have easily done loads more. - SU_06* *I was also a little bit sceptical about getting CBT, because I’d already done CBT before, I was a bit, um – and that hadn’t really helped- SU_16* *It got to the point where every time I saw her, instead of being upset and stressed about it, I was actually smiling - SU_09*
Intervention Coherence	*That way of whenever something big does come up or you feel that you need a little more emotionally drained after therapy, you’ve got tools at home you can refer back to help yourself. - SU_04* *I almost recommend that EMDRp could be, instead of just the EMDRp part where it’s the eye movement, it could literally be a – any sort of movement, really. A simple movement like drawing, painting, fidgeting with something, tapping, touching, walking, even, that – it’s a repetitive, easy motion that is reinforced by a sense of physicality. So you can feel your eyes moving back and forth, you’re watching the room move, you can feel yourself tapping against your knees or your elbows, or whatever you’re doing if you’re using that instead. - SU_03* *Things are not going to just work out very quickly, it does take time. So more people have the prediction that you’re going to go into therapy and within nine months you’re going to be cured, but I came to terms that that’s not what happens, it takes a long process. Some people it might, some people it might not. - SU_09*
Perceived Effectiveness	*she helped me get to a point where I was able to talk to other people, so it didn’t feel so lonely when I didn’t have her. - SU_09* *Yeah, it isn’t as like gut wrenching anymore. Like it’s not – I’m not breathing anymore because something is taking its toll on me. Like I can think about one and it just be, okay, that happened. - SU_06* *I do think that the therapy with RESTART has made me feel a bit more comfortable, especially with certain topics. - SU_05*
Burden & Opportunity Costs	*I knew for the rest of the day I’d be sort of drained or maudlin or potentially short tempered if a lot of anger was involved in the previous – in the therapy session. Yeah, but you’d expect it - SU_03* *It was difficult, I’m not going to lie, because, I mean, sometimes you even forgot things that had happened, and it’s only when you speak about one memory that it would unravel a few more instances where it’s happened again*, *I’d find I’m either like doing loads better or, because we’d brought stuff up, I feel a bit down. So, I think, because of what was going on at the time, it made it quite difficult to just go back on with my day - SU_06* *I felt like that was just sort of me building up to saying it. But it just made me quite anxious and like the nightmares, if I knew that I was going to have to talk about it, the nightmares did seem to get a bit worse every now and again because it’s like on my mind and stuff. - SU_08*
Self-Efficacy	*I’m grateful of the chance to rebuild my life because of it, because it’s made me wiser. - SU_10* *I was the one that was like steering the sessions a lot. Like [name] was there for like support and like the actual like therapy parts and things. But like it was very much like me doing a lot of work as well. And like I was in control of things that we spoke about and things we didn’t speak about, and I think that really helped like the whole thing. - SU_16* *I didn’t feel like there were any surprises or unexpected questions or things like that, which did help me feel very like in control, which was good. - SU_16*
Ethicality	*It just felt like an opportunity I didn’t really, couldn’t really pass up. - SU_21* *I just found it a lot more easier to have that one person knowing that that is the structure and it is the same, it’s not going to change, the face isn’t going to change, and that what I am going to say and the way that they react to it is a lot more easier. - SU_09* *It was like she understood that I – as someone from, like a certain minority group, I have certain experiences that other people might not have. SU_09*

While the analyses entailed a systematic comparison of participants’ experiences of EMDRp and TF-CBTp, and any differences were systematically explored, the reported experiences of those allocated to different trial arms were found to be homogeneous and congruent. Senior authors reviewed this finding and decided that the results would be discussed jointly in most instances. When meaningful differences appeared between trial arms, for example in participant understanding of the interventions, these are explicitly mentioned and discussed in detail below.

### Affective attitude

This theme explored participants’ attitudes towards their trauma history, therapeutic support, and the links between adversities and their current experiences. Most of the interviewees had received some form of psychological intervention previously (n=20), with just under a third receiving Cognitive Behavioural Therapy for psychosis (CBTp) from early detection and intervention services specialised in providing support for ARMS (EDIT). Over half of the sample had some experience of CBT. Only one participant had received a trauma-focussed intervention prior to RESTART.

In general, participants reported being unaware of how much their previous life experiences were impacting them before they were involved in RESTART. They were able to identify that *something was wrong* beyond what they were told or experienced but were unclear about the exact relationship between their traumatic experiences and current difficulties. All interviewees found the informal opportunity to discuss these experiences as part of RESTART assessments helpful, with participants confirming that this understanding *helped them* and there are *other people like them*, which was valued.

This knowledge led to participants questioning *whether their experience is linked to traumatic experiences* or *finally understanding* the relationship. Links between ARMS symptoms and trauma were established for some participants and reassured them that they were not *hearing [ … ] things for no reason*. Other participants maintained a clear divide between their experiences, stating that they were having *only minor psychosis-adjacent symptoms* and *everything else was directly relatable to trauma*. However, even those who did not originally link their current experiences with previous traumatic exposure, made this association as the intervention continued:

“I had no idea how badly it was affecting me [ … ] It was more the absence of the chaos that allowed me to see that the chaos was quite big to begin with”- SU_20

Alternatively, some participants stated that they understood the impact of traumatic experiences but did not feel comfortable enough to discuss it or had previously experienced negative reactions from their clinical team. Several participants described how previous clinicians often did not understand what they were going through and often told to *put it behind them* as it was *in the past*. Others felt that they were *shot down* when mentioning previous traumatic events to mental health professionals, with two participants noting that they were forced to *conceal their pain* and encouraged to focus on the *here and now:*

“I was very under supported and very introvert back then and kept things to myself too much. Didn’t speak about things, concealed my pain, and led me to self-harm and things like that”. -SU_10

Furthermore, those who had received support from an early detection and intervention team (EDIT) found it *effective and helpful* for some ARMS experiences, but indicated a strong desire for different approaches, *something new*. Specifically, interviewees felt that it was not *going in depth enough to resolve things* and thus left them *coping on [their] own.* Participants noted concerns about CBTp in Early Detection services being *surface level*, which meant that it *did not work* as it was *treating the symptom but not the root issue*. Despite these shortcomings, some participants felt that their Early Detection involvement did prepare them for future, more intense, therapeutic work focussed on traumatic experiences.

“If I hadn’t been in therapy before EDIT [Early Detection and Intervention] or before RESTART, I don’t know that I would have been as reflective and known that like I’m depressed because I’m traumatised”. -SU_20

Participants who were allocated to TAU as part of RESTART, and received therapy from other teams as part of treatment as usual, noted the positive impact of CBTp for their mental health. Interviewees noted benefit to their sleep and reactions to events alongside positive therapeutic relationships:

“She’s dug into me, my psyche and, yeah, I do believe that she understands how I think and how I feel, I really do”. -SU_12

However, all participants expressed a desire for more trauma work or focussed interventions, stating that TAU did not *really touch on the past* or did *not really work on it*. One participant noted that they hope for future intervention to:

“Help get rid of me [sic] PTSD, help me just become – help me like, you know, get to, not the old me, but the version of meself [sic], one that’s not afraid”. -SU_12

In accordance, interviewees noted that the appeal of RESTART was a *perfect opportunity* to explore their experience *in a different or more specialist* way. Alongside this, an altruistic desire to *improve outcomes for others* and put their experiences to *good use rather than just kind of keep it to myself* was present. One participant noted that they used RESTART to see if they had the ability to *navigate a discussion* about what had happened to them in the past. Above all, individuals noted being *too far gone*, at a *make-or-break moment*, and had *tried everything else* and wanted support for their mental health, even if it was minimal.

“If it’s a chance for me to even just get that slightest bit better, then I’m going to at least try it”. -SU_13

### Intervention coherence

This theme explored how much participants understood either EMDRp or TF-CBTp and what their expectations were prior to the intervention.

Those allocated to TAU and therapy arms reported some form of anxiety prior to the start of the therapy. Participants reported being either nervous, unsure or not sure if *they were ready*, particularly if they had had negative therapeutic experiences in the past. One individual noted that they did not have any anxiety as they believed they would be fine, until they started to speak about it:

“I thought that I would walk into therapy and talk about traumatic experiences that I’d thought about and be fine because I’d thought about it. [ … ] the first time I said it to [name], in the first session I was in floods of tears and I could barely get it out, and it was really heavy”. -SU_20

However, for all participants, this was explained, by them, as *normal, expected*, and *acceptable*, with some highlighting that this was particularly due to the intervention being trauma-focussed and other stated that it was just because *it was something new*. These worries did not lead to any concerns about remaining in the trial nor were there notable differences between therapy arms. Participants reported that this anxiety dissipated shortly after starting the intervention, and if it remained, this was managed and decreased over the course of the intervention.

Prior to intervention there was a stronger understanding of the philosophy and underpinnings of TF-CBTp rather than EMDRp, which was expected due to the high prevalence of participants receiving cognitive behavioural therapies from Early Detection services. Participants reported being unsure if TF-CBTp would be different from what they have received previously:

“Most of my therapy in the past has been like CBT, it hadn’t been labelled trauma-focussed CBT, so I didn’t know like if it were going to be anything majorly different”. -SU_05

However, post intervention, all participants reported an increased understanding of TF-CBTp, their own mental health and how their experiences linked their current difficulties:

“[my therapist] linked a lot of what was happening, with me hearing and seeing things, to something that was built inside of me from my mother, which helped me a lot to understand that the visual and audio stuff I am hearing is actually my body’s defence against what has happened”. -SU_09

TF-CBTp participants also highlighted the resources gained from the intervention and noted how they continue to use these following the end of their therapy:

“The resources were great to have. Like I’ve always been trying to focus on myself, try and fix the bits and have tools to help myself, like breathing exercises and meditation was what I did before therapy, and it’s definitely still good to refer back to, but it’s nice to reinforce those and get new tools. It’s giving you the tools to do therapy for yourself, I guess, in a way”. -SU_04

There was less understanding about what EMDRp was prior to the intervention, with participants noting that it is not *common to have and* felt *more specialist* than other forms of therapy. This led to anxiety from a single participant feeling like they would not understand *the science behind it* and resulted in participants doing their own research to identify what EMDRp entailed. However, this uncertainty was exciting to one participant. There was a sense from the EMDRp participants that the intervention had to be explained in more detail due to the novelty, with one participant stating:

“I was expecting it to be some sort of hocus pocus, sit and meditate sort of stuff, to be a lot more inactive than it actually was”. -SU_03

However, for both groups, when a participant expressed a desire to deepen their understanding about the intervention, this was actively encouraged and facilitated by therapists:

“she was there straight away [ … ] she facilitated the help where necessary”. -SU_10

Post-intervention, all participants reported an increased understanding of EMDRp with some reflecting on the intervention:

“I don’t think EMDRp is a very quick process, it can’t be something where you can just have three sessions and you’re good. I do think it takes some time to gain an understanding”. -SU_03

Participants reported that, to gain understanding of the intervention and how trauma impacts them, it was helpful to come up with their own metaphors in therapy. Particular emphasis was put on the work completed in sessions to aid their understanding of their past experiences, intrusions, and how EMDRp helped to process them:

“[The] nightmares and flashbacks and things, they kind of felt like a DVD that got stuck in a DVD player, that I couldn’t like stop. And then they eventually got to like being able to be out of the DVD player but not on the shelf, and then onto the shelf”. -SU_16

### Perceived effectiveness

This theme explored how effective participants found either EMDRp or TF-CBTp in reducing, or changing their perception of, unusual experiences, trauma symptoms, or other aspects of their life.

For symptoms associated with post-traumatic stress, it was reported that both EMDRp and TF-CBTp led to a reduction in experiences such as flashbacks and nightmares. Participants found that intense memories reduced in either *intensity* or *frequency*, noting that they feel *like a normal memory* or *a faded version of the same event*. Reductions in hypervigilance were also noted in both arms, as interviewees noted that they no longer *took everything as a threat* and were *able to relax.* One participant noted that engaging in therapy has made them realise that *PTSD does not control them*, and they were able to regain autonomy through this realisation:

“I kind of noticed like the decrease in like nightmares and decrease in like flashbacks and things like over time. And then, when we got to near the end of our sessions, I kind of noticed that like I felt so much different than before”. -SU_16

Some noted that the direct reduction of symptoms related to post-traumatic stress, but others expressed a reduction in emotive responses, stating that they were *more able to process the emotions* [ … ] *or more able to move on.* Participants noted that experiences became *less intense* and they *feel more confident and in control*, making them:

“More comfortable with all stuff that happened. It’s easier to talk about, it’s easier to deal with and it’s easier to remember” -SU_04

As such, interviewees felt that they were *freed* from their trauma symptoms, *unlocked emotions*, and were finally able to *speak about [ … ] and feel the emotions without it being too overwhelming or difficult.*

Participants felt that the symptoms *went away* or were *less raw* as a result of the therapy, whereas other attributed the change to having someone else to use *as a sounding board* in a *controlled environment* to process their difficult life experiences. However, this change was not immediate, as participants noted that it took time to *gain an understanding* [ … ] *to implement [the changes]*. One interviewee described this process as *chipping away* what happened to them in order to *neutralise it*.

Some noted that this reduction was due to *processing what has actually happened to them* and being able to *make connections* that they had not been able to do previously. One participant noted:

“It was definitely the most effective therapy I’ve ever had. Like I said, if I had another opportunity, I’d basically throw myself at it. It was literally – I mean, it literally fixed my life [ … ], it very much got rid of the problems and it was kind of the root problem solving that I wanted, instead of covering things up with a plaster”. -SU_20

Despite this, some interviewees did note increases in experiences associated with post-traumatic stress at certain points in the therapy, with some highlighting exacerbation at the beginning of the reprocessing and trauma memory work:

“To begin with it was horrible. [.] I wasn’t able to get through explaining the experience without crying about it or without being like – like feeling quite anxious, like I couldn’t breathe, like almost on the verge of an anxiety attack or a panic attack” -SU_20

Slight exacerbations in symptoms and distress were also reported when processing a new target memory, but some participants were able to use previous processing experience in RESTART therapy to reassure themselves:

In contrast to the positive impact on trauma symptoms, differences in ARMS experiences were less consistent, with some participants noting an improvement in the intensity and frequency of experiences, stating that:

“The symptoms of the voices and the flashbacks have actually reduced, they’re not as much. Whereas before the therapy it was all day, non-stop”. -SU_13

Others noted a decrease in conviction and belief in the source of their experiences, with participants noting an increased ability to *know the difference* between the experiences and reality and *be able to ground themselves:*

“I’m feeling better and I can just kind of push them to one side and just ignore that they’re there and get on with my day”. -SU_12

I feel as though like therapy or no therapy, I’m still going to have unusual experiences. So on paper it probably doesn’t look like therapy’s helped, but it definitely has in the way that I feel and cope with the experiences”. -SU_05

Two interviewees noted a period of ARMS symptom exacerbation while undergoing trauma work, stating that *voices got louder* and required their therapist to *monitor their own symptoms*. However, these increases were always acceptable to participants and viewed as a necessity to get better:

“It was almost like you opened up the trauma, things got a bit worse, but then when you work on them it became slightly better”. -SU_06

Interviewees noted significant changes in other aspects of their life, finding their day-to-day life *less stressful and overwhelming.* For some, it helped them *look on the bright side more* and improved *relationships* and their own *position of worth*.

For those who did not find the intervention as beneficial, this was attributed to a limitation of therapy as a whole or the length of the therapy, rather than being specific to RESTART, with one participant stating that:

“But mostly I’d say like the root – some of the other stuff which I’m depressed about, therapy can’t really help me with”. -SU_02

Despite this, the most important, and effective, aspect was the therapeutic relationship. Interviewees reported that the therapists themselves made them *feel safe, took time to believe them*, and *gave them time*. They consistently reported feeling comfortable and were always met *where they were at*, meaning that they could *talk about anything.* All participants noted feeling that their safety and support was the priority in sessions, feeling that the therapist *had everything under control*, especially when in the context of disclosure and processing of distressing traumatic experiences. Participants found the therapeutic relationship to be the most important factor in realising the therapeutic gains of the intervention, stating that the relationship helped *revive [ … ] who I am* and *gave them confidence* in the intervention and the therapeutic process. One participant noted that due to the therapeutic relationship, their experience of trauma therapy under the trial, compared with previous experiences, was:

“More effective because she was able to talk more and you’d be able to open more about your therapy and about your trauma”. -SU_06

Despite the difficult subject matter, participants were *excited* to spend time with their therapist and to *work together* in order to get better. This feeling was attributed to the consistency offered by the therapist and the effort perceived by the participant:

“One of the main common factors, not factors, like symptoms of your trauma is the fear of rejection. But with [name] her way of showing me reassurance is her wanting to continue the therapy with me, was her coming back each week to do the therapy”. -SU_13

Even the participant who withdrew from the intervention stated that:

“I stayed a lot longer than I probably would have anywhere else because of the relationship we had”. -SU_08

### Burden and opportunity cost(s)

This theme explored whether participants felt that they had to sacrifice anything to engage in the intervention, or whether they felt the intervention caused excess practical or emotional burden.

An increase in emotional burden was expected and experienced by participants over the course of therapy. Participants noted *emotional drainage* both immediately before and after sessions. This impact made them feel *wary*, *put them on edge*, and made them feel that they had the *weight of the world on [their] shoulders*. At times, this impact caused increased *nightmares* and fear of *negative memories*. These experiences were explained as the aftermath and continual *processing of experiences*.

Participants noted ongoing physical burdens when engaging in both therapies, reporting feelings of being *drained, exhausted*, and having *the wind [taken] out of you*. In sessions, participants noted that *everything would get tight* and *feel disconnected*. Both experiences meant participants needed to make space to *decompress or nap* after, to continue to engage with their lives.

Similarly, multiple practical burdens were noted, with participants needing to take *the rest of the day off* from work or university due to being *mentally tired.* This burden also caused some participants to reschedule or have shorter therapy appointments if they felt they were *not up to it*; however, in all cases, this was discussed and agreed with the therapist and adaptations were made, such as arranging appointments on certain days or times.

Despite these struggles, all participants noted that they were either expected or remained at an acceptable level. This burden *was never crippling* or made them feel *unsafe* and did *not put them off* engaging in therapy. Participants managed the burden, in collaboration with their therapists by being *more gentle* on themselves and utilising their support networks. All participants who reported a form of cost or burden indicated that their therapist was accommodating of the impact.

Participants who mentioned some form of burden or cost of therapy noted that they continued because the burden felt *appropriate, necessary*, or *enough to* manage. The key driver was the *hope that things will get better* or a *positive outcome in the end*. The potential benefit of burden within a therapeutic setting was also discussed, with one participant stating:

“I don’t think being uncomfortable during therapy is a bad thing, I don’t think therapy would be that productive if I was just constantly comfortable with everything”. -SU_05

However, future studies were recommended by participants to explicitly acknowledge that the burden is a possible side effect and to discuss this openly to establish the impact and ways of managing it:

“I think there should be some form of disclaimer, that it’s not just going to be plain sailing, you’re not going to be happy every week”. -SU_06

### Self-efficacy

This theme explored how confident participants were that they could engage with trauma-focussed interventions both pre and post intervention.

Prior to the intervention, participants reported a range of anxieties about their perceived ability to engage in the intervention, feeling *anxious*, expressing concerns about being too *scared to say anything* out of the worry of being *judged, too far gone for any help*, *getting told off*, or having the therapy offer rescinded. However, the driving force to continue with the intervention was *a chance to get better*.

However, in all but one case, this was raised with therapists, and reassurance was provided, which increased the ability to engage with the intervention:

“I thought if [name] found out what happened to me, then she might not continue with therapy, but that wasn’t the case with her”. -SU_13

The participant who did not discuss these concerns with their therapist later withdrew from the intervention. The withdrawal was also compounded by a range of practical factors alongside these concerns, such as a lack of space to confidentiality engage in therapy, as their home environment lacked sufficient privacy and were unable to travel to a clinical space. However, when asked directly about why they dropped out, they stated that it was not a concern about the therapy or the trial itself, rather:

“It was mainly just me, I just don’t think I was ready for it”. -SU_08

When asked to expand on what they did not feel ready for, the participant stated:

“I knew I was going to have to talk about things I didn’t want to. And I think in the end I just, “I’m not ready to talk about it.” -SU_08

For those who completed the intervention, self-efficacy was reported, developed over the course of the intervention. Some participants reported that while apprehension continued, they felt increasingly able to engage and gain benefit from the intervention as time went on. They reported that they were the ones *in control* and could not *believe [they] spoke about it* and that it got *easier and easier until you’re feeling okay.* While participants were appreciative and consistently highlighted the work of their therapists, they also noted that it was *me doing a lot of the work as well* and *it was all on me* to find the benefit.

Upon reflection, participants reported that RESTART was *the right thing to do* and if given the chance to undertake therapy again, they would without doubt:

“if I went to do EMDRp again it would be like going back to an old friend. Why would I not want to do EMDRp again, it fixed – it did what it needed to do”. -SU_20

Although to foster self-efficacy, interviewees noted a wide range of adaptations throughout the trial, primarily focussed on practical adaptations, neurodiversity and giving control to the participants. Specifics are provided below.

In terms of practical adaptations, participants noted the benefits of flexible appointments and arrangements. They were offered a choice of therapeutic modality (i.e., online or in-person) which was key for engagement, with interviewees noting that without the choice, they would be *really unsettle*(d) and they *don’t think [they] could have done it*. One person reported that taking part in the trial allowed them to *really personalise my [their] experience* which made them disclosure and discussion of traumatic events easier.

Participants also noted the need to feel *in control* of their experiences and have an open dialogue with their therapist about the experiences and what they wanted to work on in therapy, rather than an assumption on what memory should be worked on. This was key for one individual, who noted that:

“If it had been a very strict, “Okay, we’re going to think about this.” Say we’d picked a particular traumatic memory of something, and stuck only with that, then that would have been detrimental”. -SU_03

Having control over the process was consistently noted as required to realise the effectiveness of the intervention and desire to disclose. Explicit permission to shut down any conversations that were uncomfortable was appreciated by participants. Similarly, therapists showing genuine curiosity in who they were as a person was valued, allowing them to discuss what they felt was important without being *shunned* made participants feel that the intervention was *worth doing in the end.*

Participants who self-reported neurodiversity reported significant benefits when their experiences were explicitly acknowledged, and adjustments, such as clear and direct communication alongside allowances for extra time to process information, were made. While previous therapists had *ignored* these difficulties, the RESTART therapists took it in and considered it in formulation. Furthermore, in the EMDRp arm, one individual noted that need to have multiple techniques for bi-lateral stimulation, sensory stimuli such as eye movement performed concurrently with the recall of the worst image of the trauma, to feel the benefit, stating:

“I’ve got to feel everything. And see for me, ‘cos I can’t focus on one thing at one time, [name] gave me the buzzers as well [ … ] Yeah, so the light bar and the hand buzzers at the same time, and it’s like I just tapped into a bit of difference there”. -SU_10

Participants who reported specific symptoms identified the need for adjustments in order to effectively engage. One interviewee who described frequent dissociation reported that having written summaries of therapy sessions and key stages were helpful as:

“There were some times as well where I’d just dissociate and I wouldn’t quite hear, [ … ] so it helped that I could read it as well”. -SU_13

Another reported the need for distraction when their voices were particularly loud on certain days, noting that their therapist allowed them to keep an earphone in as:

“It does help me a lot, and it distracts my mind and the voices while I’m out and doing stuff, then I can concentrate on what I’m doing as long as I’ve got me [sic] earphone in” -SU_14

Both participants noted that these adaptions were key to them engaging and would have not received the beneficial impact of therapy without them.

In terms of adaptions for future trials, one interviewee who reported strong flashbacks when discussing their experiences, noted that they would have appreciated a non-verbal way of expressing their current state while processing:

“I think maybe having kind of a – maybe like a traffic light system of checking in of like, “Are you doing okay?” Like a green, yellow and a red. So that it’s a more explicit way of being able to measure how the processing is going in terms of like the present, of whether that person is okay or not”. -SU_20

While participants found the intervention effective and acceptable, some interviewees required adaptions in order to gain this benefit, and while the adaptions offered under RESTART, detailed previously, were helpful, they cannot be considered all encompassing. Participants should be asked individually about adaptions they feel would be beneficial in order to facilitate benefit in trauma-focussed interventions.

### Ethicality

This theme explored whether engaging in a trauma-focussed intervention fit within participants’ values and whether participants had preferences for a trauma-focussed intervention.

There was conflicting feedback regarding characteristics and attitudes of trial therapists and how that impacted effectiveness. Participants highlighted preferences for specific gender identities, particularly when specific therapist characteristics had clear links with the participants’ trauma:

“Being able to trust a woman, especially with like misogynistic experiences, felt a lot easier. I do think that if I had been assigned a male therapist, I would have tried it, but I would have been less hopeful”. -SU_20

Participants noted that if this preference was not adhered to, due to a lack of diversity among therapists, they would have not *spoken about as much as [they] did* due to *the nature of what I [they] wanted to talk about.* This preference was related to *trust* and not wanting to see *them as a threat* in some interviewees, alongside the ingrained *understanding* of some experiences without the need to explain or educate their therapist. Some participants noted a desire for therapists to share the same gender identity or class background for similar reasons, as being further away from these experiences meant that they would be less likely to *be able to understand why it was so impactful.*

However, others did not find certain characteristics *important* and did not impact *engagement or trust*. Some noted a desire for difference in their therapist or acknowledged that different therapeutic perspectives could add value:

“I could design my own therapist, why wouldn’t I want to? But I guess that kind of defeats the purpose in terms of like being in an echo chamber. If I decide my own therapist, then what they say to me and who they are as a person then kind of defeats the purpose of therapy in that sense”. -SU_20

However, participants with these preferences noted changes over the course of the trial, expressing that the positive impact of RESTART has given them more *confidence* that other people are *safe that [they] can work with now*:

“I look back now, I would engage with a male therapist now, but back then I wouldn’t, but that’s the point [name] facilitated and made that part easier, and she’s left me in a position where I actually would be okay around engaging with males now, which I think is a much healthier approach”. -SU_10

Participants valued being asked about preferences at an early stage and noted that these questions should be asked as soon as possible in future trials:

“I wouldn’t want to go through like the eligibility and then the baseline interview and that kind of thing, to find out that the entire therapy team is male and that there aren’t any female therapists for me to see and that’s the only option they have”. -SU_20

Participants who identified as being from a minoritised group noted similar experiences, in wanting a therapist from their community, but if not possible to have someone with experience of that community, and a genuine curiosity about the experiences that *other people might not have*:

“I need someone that has at least some understanding. Like if it’s not someone that is a part of the same community as me, at least someone that has been around that sort of community as me”. -SU_16

Despite these positive findings, participants mused on the limitations of trauma-focussed therapies, particularly for cumulative traumas without a defined ‘event’ such as ongoing identity targeted abuse or harassment. One participant noted that they *couldn’t just pick one event ‘cos there’s been so many things* and felt that they had *to have one traumatic event* so they can *be asked about ‘the’ event.* This participant found benefit of the intervention for a defined event but still wanted further support for these other events. Another participant who on the intervention addressed the limitations of therapy as a whole stated that the reason for their mental health struggles was that they *were poor*, which they felt that therapy could not address, highlighting the impact of socioeconomic factors in the maintenance of poor mental health.

## Discussion

This is the first study to gather a qualitative understanding of factors related to the acceptability and feasibility of trauma-focussed interventions for ARMS individuals in the context of a clinical trial. Participants were sampled from the largest clinical trial of trauma-focussed psychological interventions for those assessed as ARMS conducted to date; the RESTART trial, a feasibility RCT offering up to 24 therapy sessions of either ‘EMDRp + TAU’ or ‘TF-CBTp + TAU’ or ‘TAU alone’. The principal aims of the current study were to establish the acceptability of trauma-focussed interventions in ARMS populations alongside potential adaptions to the therapies themselves, and the trial design procedures used within RESTART.

Participants perceived both EMDRp and TF-CBTp as acceptable, effective, desired, and safe. They reported high levels of perceived effectiveness in both therapy arms, particularly for symptoms associated with post-traumatic stress. Some noted helpful reductions in ARMS symptoms; however, more commonly, participants experienced a change in their understanding of symptoms and relationship to them, noting reductions in distress and interference in functioning. Participants noted that the therapeutic relationship was fundamental to achieving the therapeutic gains they experienced over the course of treatment, and for some, this was the most important ‘ingredient’ of the intervention. Despite the positive benefits, there was significant emotional, physical, and practical burdens and costs for participants, which while distressing at points, was always seen as acceptable and tolerable.

Participants also noted a notable increase in positive aspects of their lives such as relationships with significant others. The participants linked this directly to the trauma work completed in RESTART. Participants also highlighted a decrease in experiences that could be viewed as ‘disturbances of self-organisation’ in Complex PTSD [i.e., emotional dysregulation, interpersonal difficulties, and negative self-concept, (27)], which have been shown to maintain psychotic experiences (28) and respond to trauma-focussed therapy in previous trials with participants with early psychosis (16).

Participants reported limited understandings of EMDRp compared with TF-CBTp prior to starting therapy. Participants attributed this difference in understanding to cognitive behavioural therapies being offered as part of routine care in mental health services they had accessed in the past (e.g., talking therapy services and/or early detection teams). This difference could also be attributed to the wide array of presentations for which cognitive behavioural therapies are recommended for, likely increasing the participants familiarity with this treatment approach over EMDRp. Most interviewees indicated a desire to increase their knowledge of the intervention over the course of therapy. Post-intervention, there was an increased understanding of both modalities, noting it was helpful to come up with individual understandings of key concepts, in their own words, to aid coherence and effectiveness.

Most participants in the trial described experiencing ‘self-efficacy’ over the course of the intervention, i.e., feeling like they were in control and were supported to be motivated to make the changes necessary to benefit their mental health. This developed over the course of the trial, with significant apprehension present pre-intervention, which dissipated throughout the course of therapy. According to participants, this was the direct result of the strong therapeutic relationship with their therapist. Self-efficacy was also supported through a wide range of adaptations therapists made to therapy process, including more explicit permission for participants to have control on the content and focus of therapy sessions including whether to discuss details of their traumatic experiences and collaboratively selection of the experiences to be targeted in treatment. Participants also highlighted the benefit of adjustments made to support needs related to neurodiversity, such as clear, direct communication, alongside additional time for, and different methods of, processing. Finally, interviewees felt that engaging in trauma-focussed interventions was congruent with their value systems and highlighted the importance of considering participant preferences when considering therapist allocation.

Of note, participants sometimes reported negative past experiences of psychological interventions, finding that services focussed almost exclusively on managing ARMS symptoms rather than providing broader and more holistic support that considered trauma and its psychological impacts, including its potential relationship with their unusual experiences. Consequently, several participants described not being able to understand a potential link the link between their life experiences and their current mental health difficulties prior to RESTART input. Those who regarded their mental health difficulties as being caused or aggravated by trauma felt they were not taken seriously by mental health practitioners they encountered prior to RESTART, leading to previous interventions feeling ineffective for their specific needs. However, others found that previous therapeutic work was crucial, in terms of stabilisation and readiness for further, more intensive trauma-focussed therapy, preparing them for the more in-depth trauma processing work as a part of RESTART.

The importance of the therapeutic relationship emphasised in the current findings reflects previous research that also highlighted its role as a core element of both in EMDR (29) and TF-CBT (30). In line with the views of trial participants captured in the current study, past research indicated that positive therapeutic alliance predicts trauma-related outcomes in those with PTSD (31) as well as improvements in unusual experiences, quality of life, and social functioning in both early psychosis and ARMS service users (32, 33). Our findings also mirror qualitative viewpoints documented in past studies with psychosis spectrum conditions. In particular, our findings highlighted that may participant wished to be offered trauma-focussed interventions but struggled in obtaining a referral as part of their routine care (19). They also highlighted, once therapy was initiated, the key importance of control over disclosure and selection of targeted memories (34) as well as an explicit exploration and acknowledgement of the link between trauma and current difficulties (18) as essential ingredients of effective care. Despite that trauma-focussed interventions are characterised by relatively high drop-out rates in other clinical populations (35), our findings indicating that ARMS individuals view EMDRp and TF-CBTp as highly acceptable despite the understandable emotional and practical burdens of these intensive treatments speak of the possible values of these therapies as potential adjunct to the care provided to this high-risk group, should their efficacy be demonstrated in future, suitably powered RCTs. Similarly, this desire for and perceived efficacy of trauma-focussed therapy reiterates the need for further research in this area to address the critical need for evaluation of trauma focussed interventions in ARMS populations (12).

The findings of the current study should be considered in the context of several limitations. While all participants included in our feasibility RCT were potentially eligible for participating in this qualitative study, only those who expressed interest in an interview were approached. It is therefore possible that those who completed the interview were more likely to have had a more positive experience of the interventions or the trial than those who declined to participate in this qualitative study. To mitigate this self-selection bias, purposeful sampling was utilised throughout recruitment to identify therapy clients who withdrew and/or disengaged from the interventions, as plausibly their therapy experiences could be regarded as less than optimal compared with trial participants who fully complied and engaged with the treatments offered. It is also worth acknowledging the potential impact of the main author being the interviewer in all but two cases. Due to the nature of the RESTART trial, AF completed baseline and follow-up assessments for eight individuals who later took part in the interview, potentially leading to biases in the interview and coding stages. This was mitigated by in-depth reflective logs after each interview and monitoring of the coding process by the senior researcher (KA) to ensure the findings were rooted in the data collected. The prior relationship between the interviewer and several participants was beneficial in some respects, as rapport was already built and felt by the research worker to lead to richer interview content; however, it is possible that participants may have been less inclined to share negative experiences or views about the trial or interventions as a result. To mitigate the impact of potential undue weighting of overly positive experiences, participants were always given explicit permission to share criticism and neutral or negative views about the trial and/or therapy they received and were also given different options for feeding back their views, such as changing to another interviewer, or providing written feedback on their transcript. Despite this, none of these options was taken by participants; the study team had no reason to believe that participants may be holding back certain views on their therapy and trial experiences, and the perspectives shared by study participants were nevertheless broadly positive.

The timing of the invitation to participate in qualitative interviews may also have had an impact. By approaching trial participants immediately following their final follow-up assessment, it may be that participants who found the assessment challenging, or who had ongoing difficulties, may have been less likely to agree to participate in a qualitative interview at that time. While plausible, there was no indication that this concern was present, and every reasonable effort was made to ensure that all participants had the opportunity to take part in the interview, including offering appointments at times that best suit their schedules, completing the interview using a reduced topic guide to expedite completion, and reassurance that all experiences are valued.

While a wide range of traumatic experiences, neurodivergence, and gender diversity was present, non-white groups were not well represented in the sample, which is consistent with previous qualitative research involving people with psychosis spectrum conditions (18). Understandably, this is a significant limitation on the current findings, given the need for further understanding on how minoritised communities understand their experiences, the support they find beneficial, and any potential adaptions required. Previous studies focussing on TF-CBT and EMDR for those with PTSD and other clinical populations have suggested that a ‘one size fits all approach’, without considering cultural adjustments, can hinder therapeutic gains and perceived efficacy (36, 37). Participants from these communities in the present study reported an increased understanding and the impact of certain traumatic experiences after trial interventions. Therefore, given the findings of the present study, adaptions for certain populations are be needed for EMDRp and TF-CBTp, with specifics to be established in a further coproduced study.

While purposive sampling was used to capture the experiences of minoritised groups, representation was limited due to the lack of diversity in the parent study, reflected in the limited ethnic diversity of service users accessing early intervention teams (38). To address these disparities in future research, studies need to directly recruit from service providers and pathways with established links to certain communities rather than limiting recruitment from specialist services, which has historical accessibility concerns from certain populations (38).

Several important clinical implications arise from these findings. First, like in the case of people with psychosis (39), our findings indicate that individuals with an ARMS and a history of trauma view trauma-focussed discussion and intervention essential to positive outcomes related to their mental health. Participants noted that they were not often asked about trauma as part of usual care, which may reflect both practitioners’ reluctance and unwarranted views about the willingness and ability of ARMS individuals to engage in these sensitive conversations and possibly the absence of trauma assessment and trauma-focussed therapy clinical guidelines for individuals at elevated risk for future psychosis relatively to those for first episode psychosis which already acknowledge routine trauma assessment and the provision of trauma-therapy for clients with comorbid PTSD (40). Consequently, participants either were not aware of a potential link between their previous experiences and current presentation or did not have the opportunity to discuss them and explore them with a professional. Despite this, participants still wanted to engage with a trial that specialised in trauma-focussed interventions, finding this opportunity not only acceptable, but valued. This highlights that sidelining these conversations and failing to explore trauma histories goes against participant preference and, in our sample, led to significant perceived negative impacts for participants. In line with clinical best practice for this group, our findings on the importance of the therapeutic alliance emphasise the need to prioritise and invest in early engagement phases of the interventions, as trust, psychological safety, and the client’s experiential understanding that therapists are willing and able to flex their ways of working in response to ideographic needs are essential foundations for delivering effective and acceptable trauma-focussed care for this group. Ultimately, our findings are highly supportive of the potential beneficial impacts that trauma-focussed interventions could have for this client group, and the promise of these therapies to be both safe and effective as well as a valued adjunct to routine care for the ARMS group, should future large scale clinical trial confirm their clinical effectiveness.

To conclude, the present study is the first to explore comparable trauma-focussed treatments within the ARMS population, highlighting the voices of ARMS service users and their views on adverse experiences and trauma-focussed interventions. The results indicate that trauma-focussed therapies are seen as acceptable, effective, desired, and safe from a participant perspective, which highlight the potential for the future use of trauma-focussed interventions for those assessed as ARMS in clinical services. Our findings offer a key first step in confirming the broader acceptability of the interventions considered in this study and highlights the need for future research to conduct sufficiently powered trials to establish the efficacy and clinical effectiveness of trauma-focussed interventions for those assessed as ARMS. There were significant similarities in perceived effectiveness, burden, and acceptability between both EMDRp and TF-CBTp in the current study, which has important implications for future evaluations of these interventions in ARMS populations. If the current findings on the comparability of these interventions are replicated, this could lead to greater patient choice of and accessibility of trauma-focussed interventions for ARMS participants.

## Data Availability

The raw data supporting the conclusions of this article will be made available by the authors, without undue reservation.
