# Supplementary material for: ‘Therapy or no therapy, I’m still going to have unusual experiences [ … ] but it has [helped] the way that I feel and cope.’ A qualitative exploration of service user experiences of EMDRp and TF-CBTp in the ‘REducing psychosiS risk by TARgeting Trauma‘ study
Source: Front Psychiatry. 2026 Sep 1;17:1812693. doi: 10.3389/fpsyt.2026.1812693 (PMC13574983; doi:10.3389/fpsyt.2026.1812693)
Supplement: Supplementary file 2 [file SupplementaryFile2.docx]

**Topic guide for trial participants**

**Project:** Reducing psychosis risk by targeting trauma (RESTART)**-** A feasibility clinical trial of psychological therapies for trauma in people with an At-Risk Mental State.

**Chief investigator:** Dr Filippo Varese

*What follows is a guide: -*

*The order and exact content of the questions will be determined by the participant. The topic guide may also be amended (content and order) once data collection has begun, in light of the initial interviews and consequent findings.*

**Before commencing the interview.**

1. **Begin to establish rapport with the participant** (e.g., ask them how their week / day has been and other general ‘chit chat’ to make them at ease and comfortable before starting the interview).
2. **If appropriate (if explanation about the study and consent taking has happened sometime in the past) re-introduce the purpose of interview** (e.g., “*this study looks to gather information about your experience of taking part in the RESTART trial. This is so we can understand what worked and what didn’t in the trial and what was helpful or not so helpful about the treatment you received during the trial. This is so that we can continue to learn and design the best trial we can for the next stage of the project.* Ask if they have any questions, they might have not had the chance to ask / thy did not thought of at the consent taking meeting. Take time to address any question the participant may have before asking whether they are happy to proceed
3. **Remind the participant that, whilst in most cases the interviews last about one hour, there is no set interview time** as the length depends on how much they discuss during the interview.
4. Remind the participants that they will be asked questions about their experiences, ideas and recommendations. Whilst the interview is about the impact of distressing /potentially traumatic life experiences, remind them that **they will not be asked to discuss the details** **of their traumatic events as part of the interview**, but only their views on how these experiences have affected the difficulties they are experiencing in their daily lives, and their views on the kind of psychological support that people like themselves could be offered to address these difficulties.
5. Ask the participant about their view on being asked questions from the perspective of a minority group member (if they are one). This is done to allow the participant to ‘opt-out’ of giving this perspective if this is something that is particularly sensitive to them or something they do not wish to do. However, it is important to explain to the participant why these questions are asked and what information we are trying to gather, alongside the fact that they have the power, even if they say yes to this at the beginning, they can retract this at any point. An example preamble can be found below: *We know that research as a whole, primarily recruits from and can become very focused towards majority groups in society. This means that a lot of research can directly or indirect ignore or neglect the problems and needs of certain groups in society. As such, we may ask you questions of your experience, specifically from the perspective of (insert minority group here). The reason we ask this is because we believe it is important that RESTART and any other future research trials best represent the views and needs of your group, alongside making sure that the interventions offered by our studies can be adapted for the specific needs required. You are under no obligation to answer these questions and* *if you wish to skip any, please let me know.*
6. **Reassure the participant that they can stop the interview at any point**, either temporarily (for a break) or withdraw completely.
7. **Remind the participant that the interview will be audio-recorded**, and that this is necessary in order to make sure we do not miss important information and advice they will provide, and to ensure a full analysis of the data. Ask explicitly if the participant has any concerns in relation to being recorded or about how the recording will be handled and take time to address any concerns (e.g. reassure them about the processes used to ensure that the data will be handled confidentially and securely; show them the Dictaphone and how it works; if the interview is conducted though remote means, explain how this will work; have a trial run at recording etc.). Reassure them that whilst many people do not particularly like the idea of being recorded, most participants soon forget about the Dictaphone / the recording as they get talking. However, if participants think they will find this disturbing, collaboratively explore ways in which the process could be made more acceptable to them (e.g., certain participants may have specific fears of technology and be unsettled by a recording device in the room, but they might find it easier to engage in the process if certain arrangements are made e.g. if the device is not directly in their field of vision for example, or if they are shown how the recording can be stopped; remember that every person is different so these options will not work for everyone).
8. Remind them that **any personally identifiable information or other information that may identify them will be redacted out at the transcription stage**, to protect their privacy and confidentiality, and ensure that the participant knows that **they do not have to discuss anything they do not want to.**
9. **Ask which terminology they prefer around the topic at hand**. For example, trauma or distressing life events - Whichever phrasing they prefer can then be used throughout the interview
10. If the interview is conducted via remote means, ensure that the participant has **access to a space where they won’t be disturbed or overheard** in the subsequent hour. Make sure that, ahead of starting the interview, you have agreed with the participant a plan to follow in case the communication/call is interrupted or there is an emergency (i.e. agree an alternative mode of contact; ask for specific information on where they are; the contact details of a next of kin and permission to contact the nominated person in case of an emergency)/
11. **Make the participant aware that in some cases confidentiality can be broken**. For example, if there is a significant risk to self or others- there is a duty to take this information further in order to ensure everybody is safe.
12. Ask if they are comfortable to begin, and reassure them that if any point they wish to stop they can let you know

**Starting the interview.**

1. **Start the recording** and remember to ‘label’ the recording (e.g., “*This interview with participant [Participant ID code] was conducted by [name and surname of research worker] on [date of the interview]*”). If for whatever reason the recording must be stopped (as opposed to be paused) and if when restarted another recording (i.e. file) will be produced, remember to label any additional file/recording as above.
2. **Start with asking demographic questions included in the demographic questionnaire** e.g. **“***if you feel comfortable to begin, I will start by asking some general information about you, for example your age, your education etc.*” This provides an opportunity for the participant to ‘break the ice’, get used to the interview and accustomed to being recorded before proceeding to topics that might be potentially more sensitive and emotional for them
3. The interviewer will then **transition to the main questions of the interview** e.g. “*if you feel comfortable to continue, I will start by asking some questions about any experiences you have had of accessing therapy*.”

**Prompts and probes**

These may be used throughout the interview where appropriate, for example when encourage participants to elaborate further, demonstrating curiosity and encouraging further exploration of topics or gauge their willingness to continue with the interview:

- Please remember there are no right or wrong answers.
- Can you elaborate on that?
- How did that make you feel?
- How do you feel about it now?
- Do you feel okay to continue?
- Is there anything you would like to add?
- Please could you clarify what you meant by…?
- So, what you are saying is ‘paraphrase what the participant has told you’… is that correct?
- That’s interesting… Can you tell me more about that?
- If the interviewer misunderstands, participant can correct them by saying ‘that is not what I meant’

**Demographic Information**

| What is your age / How old are you? |  |
| --- | --- |
| How would you describe your gender?  What are your Pronouns? |  |
| Does your current gender identity match your gender identity assigned at birth? |  |
| How would you describe your sexual orientation? |  |
| How would you describe your ethnicity? |  |
| How would you describe your relationship status? (Are you in a relationship / do you have a boyfriend or girlfriend?) |  |
| Do you work/study currently? How would you describe your employment status? |  |
| What is your highest completed level of education? |  |
| Do you consider yourself to have a disability? If so, what are your disability/disabilities? |  |
| Are you currently being supported by mental health services? What services are you receiving support from? |  |
| Prior to taking part in the RESTART trial, had you had any prior experience of Psychological Therapy? |  |
| Have you ever been admitted to hospital because of a mental health problem? What was that for? How many times has this happened? |  |
| Has a doctor or another mental health professional ever told you have a specific psychiatric diagnosis? What was this? When were you told this and by whom? |  |
| Are you taking any medication for mental health problems? If so, can you tell me what you are taking and what dose? |  |

**Section one - Feelings before being referred to the trial**

*These questions will ask you a bit about how you became involved in the RESTART trial.*

Can you remember how you found out about the RESTART trial?

Did you feel you had plenty of information about what the trial involved before starting?

How did you decide to become a part of the trial?

How did you know that the trial would be suitable for you and your experiences?

- Did you feel that your experiences fell under the label of trauma/stressful life events? Why/Why not?
- Is there any particular phrasing or action that has helped/would help with this?
- Did you feel safe to talk about this within RESTART? What about other teams?

What expectations did you have when starting the trial?

- How did you feel about taking part in a trial of this kind?

What were your thoughts about being offered a trauma therapy?

- Prompt/follow-up: Did you have any concerns?

**Section two – Experiences of support**

*I’ll now ask you some questions about your experiences during the trial and the support you have gotten in the last nine months, please remember there are no right or wrong answers.*

Are you still receiving support from your mental health team?

- If YES- what support are you receiving? How often are you receiving this support?
- If NO- How did you feel coming to the end of the support you were receiving?

IF ALLOCATED THERAPY WITH MH TEAM:

How did you find your relationship with your therapist?

- Were you able to connect with your therapist? Why/why not? Did you feel able to bring this up to them? If so: What happened?
- Do you feel your therapist was able to understand how your experiences impact you on a day to day basis?
- How much do you feel they were able to offer a solution for what has happened in the past?

Was there anything that the therapist themselves did that you found helpful or not so helpful? What did they do that made the biggest difference?

What did you find the most important part of therapy was?

- Was there a part of therapy that was particularly effective for your specific difficulties?
- Was there a part of therapy that was particularly helpful for your mental health as a whole?

Did the therapy focus on or include any work on your difficult life experiences?

- IF YES:
- How did you find talking about these experiences?
- Was there a part of therapy that was particularly effective for your specific difficulties?
- How did you manage the impact of talking about these experiences?
- Was the therapy relevant to the way trauma affects you?
- Has the way you feel about your trauma/difficult life experiences changed since having therapy?

How effective did you feel the intervention was?

- How did the intervention impact on your symptom(s)? What symptoms? When? How much? Did anyone else notice? When/How?
- How did this change over time? (i.e. was it better/worse during or after therapy)
- Whether the therapy changed how you cope with these symptoms when they do come up?
- After therapy, do you think your traumatic events will continue to impact you? How? What can help?
- What has the impact been on your day-to-day coping?
- Has therapy had an impact on any other conditions/experiences/aspects of your life?

What do you find is the most important part of support you **[receive/received]** from your mental health team?

Have you found the support you’ve had over the last nine months helpful?

- Prompt/follow-up: Was there anything different you would have liked, e.g. a certain type of therapy?

Has your life changed in any way since having the support from your MH team **OR** how do you hope things might change due to the support you are having from your MH team?

- Prompt/Follow-up: relationships / work / meaningful activity / social / recreational / physical or mental health / level of other treatments used (GP visits / use of medication / street drugs etc?) Can you give me an example?

Has the way you feel about your trauma/difficult life experiences changed **[since/during]** the support you’ve **[had/are having]** from your mental health team? (language dependant on participant preference)

Have your coping strategies changed in any way **[since/during]** the support you’ve **[had/are having]** from your mental health team?

**IF ALLOCATED TO TAU GROUP:**

How did you find the allocation phone call?

- How did you react? Did you feel prepared?
- Did you feel you had adequate support offered to you during this call? Did you know who to reach out to if you felt like you needed extra support?
- Is there anything we could do differently in the future?

**Section three: Trial participation**

*In this section I’ll ask you some questions about your experience of taking part in a research trial, this is so we can learn and continue to make research trials a good as they can be. Obviously, we want your honest answers so please do not worry about upsetting us. However, if you would prefer to provide answers to another member of the team, please let us know and this can be rearranged.*

How did you find taking part in the research trial?

Prompt/follow-up: Was there anything you really like or didn’t like?

How did you find the process of interacting with a blinded researcher?

- Was this easy/difficult to understand?
- How did you maintain the blind?
- Is there anything we can do differently in the future?

How did you find interacting with the research staff?

How did you find completing the measures with research staff, for example, the trauma questionnaire?

- How did you find the length of the assessment and follow-up sessions? Did this have a positive or negative impact on your participation in the trial?
- Were there any measures that were particularly difficult?
- Did you feel your experiences were captured by the trauma questionnaire? Why/Why not? What experiences were not captured? How do you feel we could capture these in the future?

How did taking part in the trial impact your support from your clinical team?

- Did your clinical team offer support throughout the course of the study? If not would this be helpful?
- Would the RESTART team having closer links (i.e. regular meetings/catch-ups) with your clinical team had an impact on your experience of the trial?

Is there anything you think we could have done differently?

**Section five: Future trial**

*These questions will ask what you think of what we could do in future trials.*

How do you think we would know if therapy was working for someone?

- Prompt/follow-up: do you have any ideas about how we could measure this or do this differently?

If therapy wasn’t working for someone, do you think it would be a good idea to stop that therapy and try a different one?

- Prompt/follow-up- Can you think of any problems with this?
- Prompt/follow up- if YES- do you have any ideas about how we could address those problems?
- If offered the opportunity to change therapies, would you? Why/Why not?

[If endorsed any of the previous questions related to minority group status] Is there anything you feel we could do differently to make the trial more accessible for people with similar experiences to you?

- Accessible in terms of community outreach (i.e. how can we better reach your communities that are underrepresented in research?)
- Accessible in terms of actually supporting the individual needs of this group (i.e. Was the trial/treatment adjusted in any way to best suit your needs/experiences?)
- IF SO: How was this done/how can we make sure this is done in the future
- IF NOT: How could future trials be adjusted to make sure it is more accessible?

Is there anything else you think we, or other trials, could do differently in the future?

**Closing section**

**Check in with the participant.**

- How do you feel the interview went?

**Check in about content.**

- Is there anything else you would like to add?
- Is there anything you feel you didn’t get the chance to discuss?

**Thank the participant for taking part.**
